# A Scoping Review of Prevention Classification in Mental Health: Examining the Application of Caplan’s and Gordon’s Prevention Frameworks (2018–2024)

**DOI:** 10.1007/s10935-025-00834-1

**Published:** 2025-03-20

**Authors:** Johannes Stephan, Jan Gehrmann, Monika Sinha, Ananda Stullich, Frank Gabel, Matthias Richter

**Affiliations:** 1 Department Health and Sport Sciences, Chair of Social Determinants of Health, TUM School of Medicine and Health, Technical University of Munich, Munich, Germany; 2 Department Clinical Medicine, TUM School of Medicine and Health, Institute of General Practice and Health Services Research, Technical University of Munich, Munich, Germany; 3 Department Prevention and Rehabilitation, RehaPro Implementation Consultant for Cooperation and Joint Projects, German Pension Insurance (Bund), Berlin, Germany; 4 Unit Rehabilitation Strategy and Social Medical Service, Department Rehabilitation Strategy and Medical Rehabilitation Facilities, German Pension Insurance Central Germany, Halle, Germany

**Keywords:** Mental health, Prevention framework, Risk-based prevention, Disease-stage-based prevention, Primary prevention, Secondary prevention, Tertiary prevention, Universal prevention, Selective prevention, Indicated prevention, Targeted prevention, Caplan, Gordon

## Abstract

Mental health prevention is a global priority owing to the increasing burden of mental disorders exacerbated by global crises such as the COVID-19 pandemic, climate change, economic instability, and armed conflicts. These crises have heightened the need for effective preventive strategies addressing mental health across different life stages and populations. To structure and classify such strategies, Caplan’s and Gordon’s frameworks have been widely used, with one focusing on disease progression and the other on population risk. Although both frameworks are frequently used in mental health prevention, their application in clinical trials remains unexplored. This review addresses this gap by examining how Caplan’s and Gordon’s frameworks have been applied in mental health prevention, identifying research gaps, and exploring their potential for their combined application to enhance prevention strategies. A scoping review was conducted following PRISMA-ScR guidelines. Studies were selected based on predefined criteria and the data were synthesized. The search spanned PubMed, Scopus, APA PsycArticles, and PubPsych, covering peer-reviewed clinical trials, including randomized controlled trials, published between 2018 and 2024 in English or German. Eligible studies classified interventions based on Caplan’s framework, which focuses on disease stage (primary, secondary, tertiary), or Gordon’s framework, which categorizes prevention by population risk (universal, selective, indicated). Studies had to focus on mental health prevention, include populations relevant to mental health and well-being, and report mental health or well-being outcomes. Of the 40 included studies, six applied Caplan’s framework, 30 applied Gordon’s framework and three used a modified classification based on Gordon’s approach. One study applied both frameworks, highlighting that their complementary use is rare. Studies were conducted in 19 countries, with the highest number from Germany (n = 8), the USA (n = 8), and the Netherlands (n = 6), across four continents (Asia, n = 5; Australia, n = 5; Europe, n = 22; North America, n = 8). Gordon’s framework was applied more frequently, particularly in universal (n = 15) and indicated prevention (n = 12), while Caplan’s framework was used mainly in primary prevention (n = 4). Depression (n = 25), anxiety (n = 21), stress (n = 8), and general mental health (n = 8) were the most frequently assessed outcomes. The studies targeted diverse populations, including children (n = 7), adolescents (n = 8), children and adolescents (n = 1) parents and their children or adolescents (n = 2), university students (n = 6), working adults (n = 7), older adults (n = 1), and adults without specifying (n = 8). This review highlights the underutilized potential of integrating Caplan’s and Gordon’s frameworks in mental health interventions. Two application examples illustrate how these frameworks can be combined to structure prevention strategies more effectively. Future research should explore combining these frameworks to enhance prevention strategies and address the emerging global health challenges.

## Introduction

Mental health prevention has become a critical public health priority due to the rising global burden of mental disorders, exacerbated by recent events such as the COVID-19 pandemic, the climate crisis, financial crises, and armed conflicts (Chakravorty, 2022; Kurapov et al., 2023; Santomauro et al., 2021; Talamonti et al., 2024; Thoma et al., 2021). Mental health conditions, including depression, anxiety, and stress-related disorders, significantly contribute to the global disease burden, leading to long-term social and economic consequences (Campion et al., 2020; Vadivel et al., 2021). As mental health issues continue to increase, there is an urgent need for effective preventive strategies that address these disorders across different stages and population groups. Two foundational frameworks for classifying preventive measures, Caplan (1964) and Gordon (1983), have provided a structured approach to shaping mental health interventions. The classification of preventive interventions, which encompasses both behavioral and structural preventive measures, either individually or in combination, is crucial for reaching an appropriate target group and establishing relevant outcomes that determine the effectiveness of interventions (Compton & Shim, 2020; Cowen, 2000).

Caplan’s framework classifies prevention into primary, secondary, and tertiary stages, focusing on disease progression. Primary prevention targets individuals before symptoms arise (e.g., mental health education); secondary prevention involves early detection and intervention (e.g., screening for early signs of depression); and tertiary prevention aims to manage established conditions (e.g., rehabilitation for chronic mental disorders) (Caplan, 1964; Duclos, 2020). Caplan’s framework has been applied extensively in mental health care. However, it has been criticized for its rigid stage separation into primary, secondary, and tertiary prevention, which assumes distinct boundaries between prevention efforts and may not fully accommodate the fluid and overlapping nature of mental health conditions, as well as its reliance on the biological origins of disease and the implied hierarchy between primary and secondary prevention, potentially misguiding public health priorities (Gordon, 1983; Mrazek & Haggerty, 1994).

Gordon’s framework categorizes prevention based on population risk levels, distinguishing between universal, selective, and indicated interventions. Universal prevention, such as broad mental health awareness campaigns, targets the general population and, therefore, focuses on the same target group as primary prevention. Selective prevention focuses on at-risk groups, such as adolescents from disadvantaged backgrounds, whereas indicated prevention targets individuals who either display early symptoms of mental health issues or are exposed to established risk factors (Franzkowiak, 2022; Gordon, 1983). Although Gordon’s framework is praised for its effective application in public health risk stratification, it may face challenges in mental health, where interventions often require a more nuanced and patient-specific approach. Broad population-level strategies can struggle to accommodate the complex and multifaceted nature of mental health conditions (Patel et al., 2018).

Building on Gordon’s classification, an extended framework consolidates selective and indicated prevention into a single category, ‘targeted,’ while maintaining the distinction between universal and risk-based approaches (Fjermestad et al., 2020; Scalora et al., 2022). This modification simplifies Gordon’s original classification and aligns with broader public health strategies (Mrazek & Haggerty, 1994).

In addition to Caplan’s and Gordon’s frameworks, the Mental Health Intervention Spectrum, developed by the *Institute of Medicine (IOM) Committee on Prevention of Mental Disorders* (Mrazek et al., 1994), offers an integrated and widely recognized framework that addresses prevention, treatment, and maintenance. While it emphasizes a continuum of care, its broad focus on transitions across these stages makes it less suited for a detailed examination of prevention as a distinct concept. In contrast, Caplan’s and Gordon’s models explicitly differentiate prevention strategies, making them particularly relevant for assessing preventive approaches in clinical trials.

By integrating Caplan’s disease stage-based and Gordon’s risk-based frameworks, preventive measures can be more precisely tailored to target populations and health outcomes. Despite their conceptual differences, they offer complementary perspectives that enhance intervention timing and risk stratification. This combined approach enables more effective targeted interventions and improves resource efficiency (Denisova et al., 2020; Fusar-Poli et al., 2021; Gail & Pee, 2020; Kraus et al., 2024).

Several studies have explored preventive strategies in mental health interventions (Budd et al., 2021; Colizzi et al., 2020; Fehily et al., 2020; Fusar-Poli et al., 2021), but there have been no comprehensive reviews specifically examining the application of Caplan’s and Gordon’s prevention frameworks in clinical trials, indicating a gap in understanding how these frameworks have been applied or integrated.

To address this gap, this scoping review systematically examines how Caplan’s and Gordon’s prevention frameworks have been applied in mental health interventions and explores their potential integration. It focuses on clinical trials, including but not limited to randomized controlled trials, conducted between 2018 and 2024. The selected timeframe reflects shifts in mental health prevention strategies driven by the global impact of the COVID-19 pandemic and the increasing demand for adaptable mental health interventions (Campion et al., 2020; Vadivel et al., 2021). By analyzing the application of Caplan’s and Gordon’s frameworks, this review identifies trends, geographic distribution, and opportunities for their integration, offering insights into prevention strategies and informing future public health interventions.

## Method

This scoping review was conducted between June and August 2024 to explore how preventive measures in mental health are classified in the literature. This review followed a structured process, including identifying, selecting, and analyzing relevant studies. The PICo framework, which stands for Population, Intervention, and Context, guided the search strategy, ensuring a systematic and comprehensive identification of relevant studies (Stern et al., 2014). Unlike the PICO framework, which is primarily used for evaluating intervention effectiveness, PICo is more appropriate for this scoping review as it facilitates the classification of preventive measures without requiring direct intervention comparisons. This approach aligns with the objective of mapping preventive strategies rather than evaluating their impact. No formal protocol was registered for this scoping review.

The PICo framework was operationalized through defined criteria for Population, Intervention, and Context. The Population included individuals receiving interventions aimed at improving mental health and well-being, incorporating terms such as ‘mental health,’ ‘well-being,’ ‘emotional health,’ ‘psychological health,’ ‘psychiatric health,’ ‘burnout,’ ‘stress management,’ ‘life satisfaction,’ and ‘depression.’ The Intervention component focused on prevention programs classified by Caplan’s (primary, secondary, tertiary) or Gordon’s framework (universal, selective, indicated). To ensure comprehensive coverage, additional terms such as ‘Caplan,’ ‘Gordon,’ ‘global prevention,’ ‘targeted prevention,’ and ‘prevention classification’ were included. The Context was limited to clinical trials and randomized controlled trials published between 2018 and 2024, using search terms such as ‘clinical trial,’ ‘randomized controlled trial,’ and ‘peer reviewed’.

The literature search was conducted in June 2024 using PubMed, Scopus, and EbscoHost (including APA PsycArticles and PubPsych), which were chosen for their comprehensive coverage of clinical trials, psychological research, and interdisciplinary health studies. Grey literature sources such as clinical trial registries, dissertations, and conference proceedings were not included due to resource constraints and to maintain a focus on studies that have undergone peer review. An initial exploratory search revealed that searching without quotation marks (“”) retrieved articles that merely mentioned the term “prevention” without categorizing the preventive measures into any framework, such as those of Caplan or Gordon. To enhance the specificity of the search and ensure that retrieved articles explicitly applied the preventive frameworks of interest, quotation marks were used around key phrases (e.g., “Primary Prevention”). This strategy limited the search results to articles that specifically used the exact phrases, reducing irrelevant hits and increasing the relevance of the findings. Furthermore, search terms were restricted to the [Title/Abstract] fields to improve the efficiency and manageability of the search process, which is appropriate for scoping reviews aiming to map existing literature within practical constraints (Peters et al., 2015). Limiting the search to titles and abstracts focuses on articles where the key concepts are prominently discussed, ensuring that the most relevant studies are identified without generating an unmanageable volume of literature (Levac et al., 2010).

The search strategy included the following terms: *((“Primary Prevention”[Title/Abstract] OR “Secondary Prevention”[Title/Abstract] OR “Tertiary Prevention”[Title/Abstract] OR “Universal Prevention”[Title/Abstract] OR “Selective Prevention”[Title/Abstract] OR “Indicated Prevention”[Title/Abstract] OR “Prevention”[Title/Abstract] OR “Caplan”[Title/Abstract] OR “Gordon”[Title/Abstract] OR “Global Prevention”[Title/Abstract] OR “Targeted Prevention”[Title/Abstract] OR “Prevention Classification”[Title/Abstract]) AND (“Mental Health”[Title/Abstract] OR “Burnout”[Title/Abstract] OR “Depression”[Title/Abstract] OR “Stress Management”[Title/Abstract] OR “Life Satisfaction”[Title/Abstract] OR “Work Ability”[Title/Abstract] OR “Well-being”[Title/Abstract] OR “Psychological Health”[Title/Abstract])) AND ((clinicaltrial[Filter] OR randomizedcontrolledtrial[Filter]) AND (2018:2024[pdat]))*. Boolean operators were used to connect keywords within each category using “OR” and to combine different categories using “AND.” This structured approach ensured the search was focused and relevant to the research questions while maintaining a manageable number of results suitable for a scoping review.

### Research Question and Sub-Questions

The main research question guiding this review is: *How have Caplan’s and Gordon’s prevention frameworks been applied in mental health interventions in clinical trials published between 2018 and 2024 and what trends can be observed in their application?*

Five sub-research questions were formulated to address specific aspects of this application and to provide a detailed understanding: How frequently are Caplan’s and Gordon’s frameworks applied in clinical trials and randomized controlled trials for mental health prevention interventions? Are there differences in the application of these frameworks across various specific areas of mental health (e.g., general mental health, burnout, and stress management)? Are certain frameworks more commonly applied in specific geographic regions? Have there been temporal trends in the application of Caplan’s and Gordon’s frameworks over the past five years? Are there differences in the demographic characteristics (e.g., age and sex) of the populations targeted by interventions applying Caplan’s and Gordon’s framework?

### Study Selection

Two independent reviewers (JS and JG) conducted the study selection process at all stages, including title, abstract, and full-text screening. Any discrepancies were resolved through discussion, with a third reviewer (AS) available for consultation if needed. However, consultation was not required.

The initial search yielded 401 articles, which were exported to Citavi 6.18 for reference management and deduplication. After removing 74 duplicates, 327 articles remained for title and abstract screening and were assessed against the predefined inclusion and exclusion criteria. Following this process, 65 articles were selected for full-text review. One article was requested from the authors, but no response was received.

**Fig. 1:**
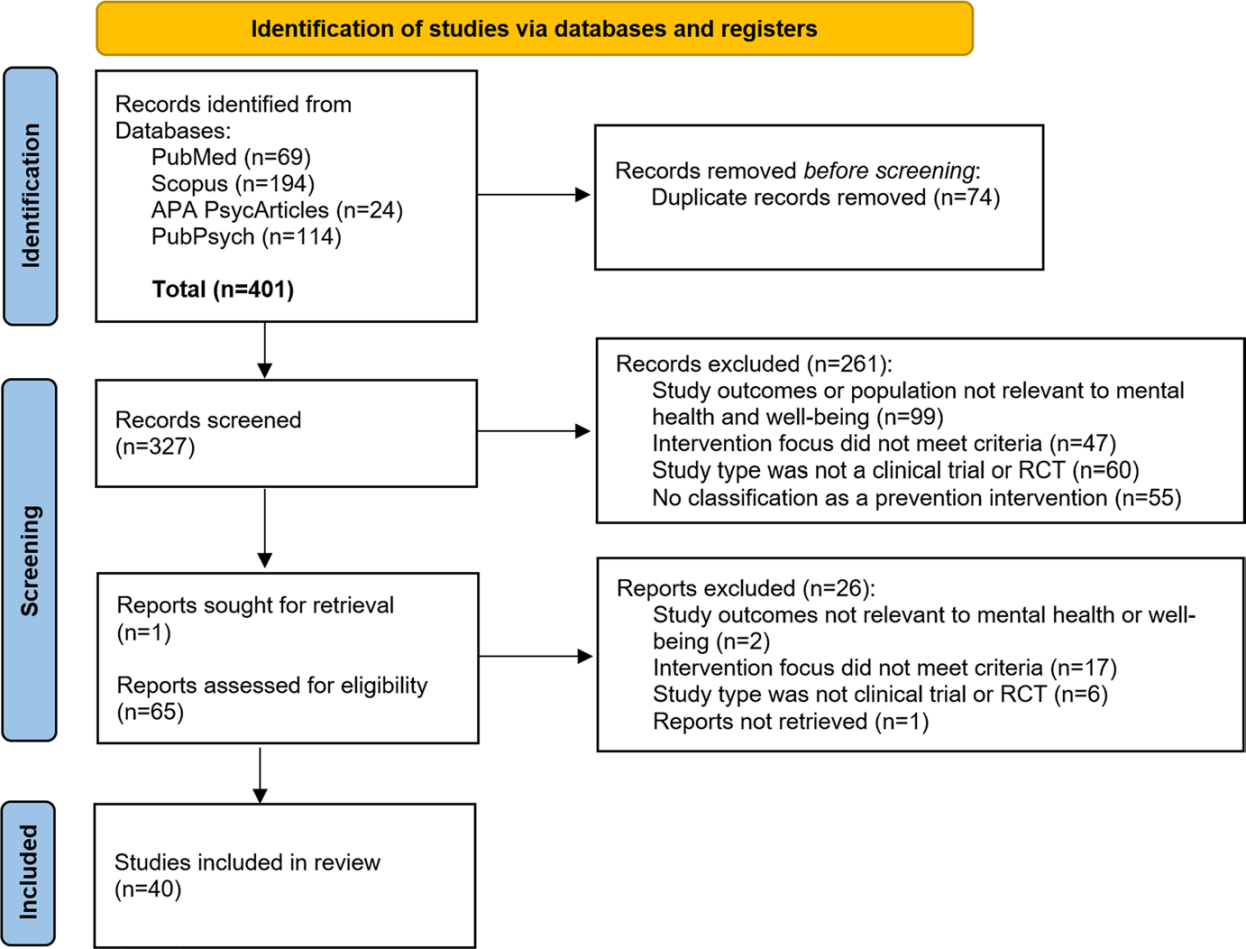
PRISMA flow diagram following Page et al. (2021)

After full-text review, 26 articles were excluded for the following reasons: the outcome was not relevant to mental health or well-being (n = 2), the intervention did not meet the criteria for preventive approaches in mental health or was not classified under Caplan’s or Gordon’s framework (n = 17), the study was not a clinical trial or randomized controlled trial (n = 6), or the full text was unavailable (n = 1). Ultimately, 40 studies remained and were included in the final analysis. The study identification, screening, and inclusion process is summarized in the PRISMA flow diagram (Fig. 1), which outlines the number of articles retrieved, screened, and excluded at each stage.

### Inclusion and Exclusion Criteria

To ensure alignment with the research questions and the focus on mental health prevention frameworks, studies were included if they were published in English or German, peer-reviewed, and published between 2018 and 2024. Only clinical trials, including randomized controlled trials, were considered, provided they classified interventions according to Caplan’s framework (primary, secondary, tertiary) or Gordon’s framework (universal, selective, indicated). Furthermore, studies had to focus on interventions aimed at promoting and enhancing mental health, including populations relevant to mental health and well-being, and report mental health or well-being outcomes.

Studies were excluded if they were published before 2018, were not peer-reviewed, or were not written in English or German. Non-clinical trials were also excluded, as well as studies that did not classify interventions according to Caplan’s or Gordon’s frameworks. Research involving populations not relevant to mental health and well-being or failing to report mental health or well-being outcomes was excluded. Studies focusing on dementia prevention or suicide prevention were excluded to maintain alignment with the review’s scope. While some suicide prevention strategies, such as awareness campaigns, align with primary or secondary prevention, many others focus on crisis intervention, which extends beyond the scope of Caplan’s and Gordon’s models. Therefore, studies focusing solely on suicide prevention were excluded. However, studies incorporating suicide prevention within broader mental health strategies were included, if the primary focus remained on general mental health outcomes.

### Data Charting, Assessment, and Synthesis

Data were charted from the 40 included studies using a standardized template developed following the *recommendation for the extraction, analysis, and presentation of results in scoping reviews* (Pollock et al., 2023). The charting process focused on capturing key characteristics across all studies to ensure a systematic and consistent approach. Categories included: author, title, publication year, digital object identifier (DOI), language, journal name, country or region, continent, study design, sample size, population demographics (e.g., age, gender, socioeconomic factors), population group (e.g., children, adults, healthcare workers), mental health focus (e.g., anxiety, depression), the framework applied (Caplan’s or Gordon’s), type of prevention (e.g., primary, secondary, tertiary, universal, selective, indicated), intervention description (providing details on the components, duration, and frequency of the intervention), implementation strategies (how the intervention was carried out, including the setting and any facilitators or barriers), reported outcomes (the specific mental health outcomes measured), and results (whether the intervention demonstrated significant effects, such as reductions in symptoms or improvements in well-being). To categorize studies under Caplan’s or Gordon’s frameworks, we analyzed the context and application of prevention terminology within each study. Studies were assigned to a framework based on the correct application of terms consistent with Caplan’s stages (primary, secondary, tertiary) or Gordon’s risk levels (universal, selective, indicated). Explicit citation of Caplan or Gordon was not required; instead, the focus was on how accurately the studies applied these concepts in their interventions and descriptions. This approach ensured that studies were classified based on substantive alignment with the frameworks rather than mere citation. Two reviewers (JS and JG) independently conducted data charting, with discrepancies resolved through discussion. To ensure consistency, both reviewers reviewed a sample of five studies before the complete charting process to calibrate the approach.

Qualitative and quantitative syntheses were performed to explore how Caplan’s and Gordon’s frameworks were applied across the selected studies. This scoping review sought to map trends, patterns, and research gaps in the application of these frameworks rather than assess the interventions’ effectiveness. Thematic analysis (Braun & Clarke, 2006) was employed to identify common themes and variations in the application of these frameworks, while quantitative analysis focused on the frequencies of each prevention framework and its respective types, along with the geographic and temporal distribution of studies.

As this is a scoping review, a formal critical appraisal of study quality was not conducted. The goal was to explore the application of prevention frameworks, not to assess methodological rigor or risk of bias (Arksey & O’Malley, 2005; Page et al., 2021; Peters et al., 2024; Pollock et al., 2023). Therefore, no studies were excluded based on quality, and all studies meeting the inclusion criteria were included for analysis.

## Results

### Distribution of the Application of Caplan’s and Gordon’s Frameworks

**Fig. 2:**
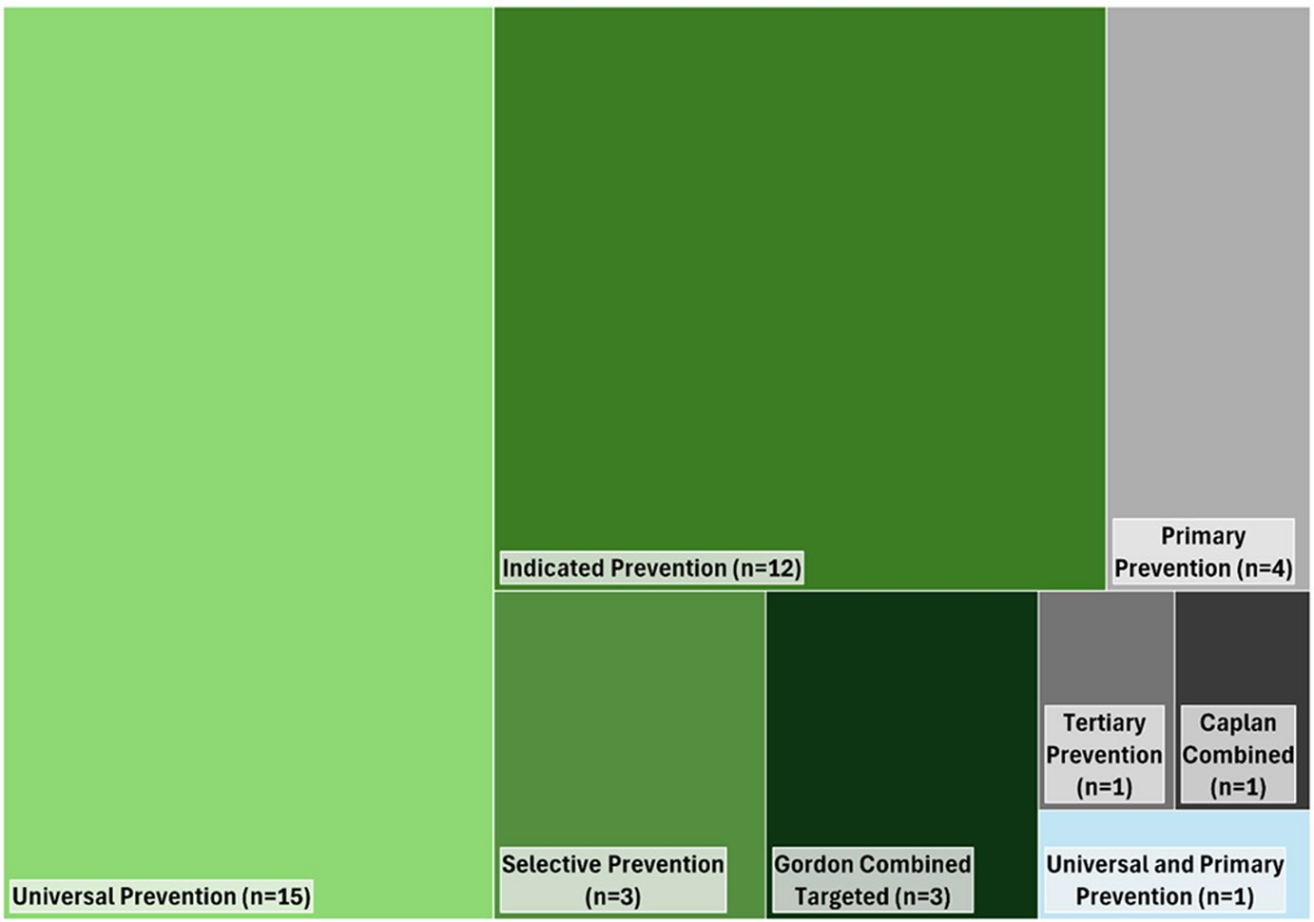
Graph map of the distribution of Caplan’s and Gordon’s prevention frameworks and types

Of the 40 included studies (see Table 1) six applied Caplan’s framework (primary, secondary, and tertiary prevention), 30 applied Gordon’s framework (universal, selective, and indicated prevention), and three utilized a classification based on Gordon’s framework (universal and targeted prevention). One study applied both frameworks, highlighting that their complementary use is rare. Figure 2 presents the distribution of studies that applied Caplan’s and Gordon’s frameworks, including the specific types of prevention. The studies classified under Gordon’s framework are represented in green tones (universal, selective, indicated, and targeted prevention), while those under Caplan’s framework are in grey tones (primary, secondary, tertiary, and combinations of these types of prevention). The study that applied both frameworks is represented by a blue tone. This visualization illustrates the frequent use of Gordon’s risk-based framework in mental health prevention studies, particularly in the universal and indicated prevention categories, while also depicting the less common application of Caplan’s stage-specific framework.

**Table 1:** Included studies for the analysis

Study ID	Author(s) [Source]	Publi-cation Year	Applied Framework	Type of Prevention	Country	Evaluated Outcome	Studied Population
1	Ahlen et al.	2018	Gordon	Universal Prevention	Sweden	Anxiety, depression	695 children (age 8–11)
2	Cardamone-Breen et al.	2018	Gordon	Universal Prevention	Australia	Anxiety, depression	349 parents, 327 adolescents (age 12–15)
3	Havermans et al.	2018	Caplan	Primary and Secondary Prevention	Netherlands	Anxiety, depression, stress, work-stress	473 working adults (healthcare workers)
4	Schoneveld et al.	2018	Gordon	Indicated Prevention	Netherlands	Anxiety	174 children (age 7–12)
5	Ahlen et al.	2019	Gordon	Universal Prevention	Sweden	Anxiety, depression	695 children (age 8–11)
6	Beaudry et al.	2019	Gordon	Universal Prevention	USA	Depression, mental health literacy	201 adolescents
7	Coudray et al.	2019	Gordon	Indicated Prevention	USA	Anxiety, depression, stress	782 university students (age 18–21)
8	Dias et al.	2019	Gordon	Indicated Prevention	India	Anxiety, depression	21 older adults (age + 60)
9	Makover et al.	2019	Gordon	Indicated Prevention	USA	Anxiety, depression	497 adolescents
10	Weisel et al.	2019	Gordon	Indicated Prevention	Germany, Switzerland, Spain, Netherlands	Anxiety, depression	954 adults (aged + 18)
11	Esaki et al.	2020	Gordon	Selective Prevention	Japan	Depression	683 working adults
12	Fjermestad et al.	2020	in association to Gordon	Targeted Prevention	Norway	Anxiety, conduct problems, depression	170 children and adolescent (age 8–16)
13	Games et al.	2020	Gordon	Universal Prevention	Singapore	Anxiety, depression, stress, resilience, self-esteem	76 university students
14	Kliem et al.	2020	Gordon	Universal Prevention	Germany	Well-being, self-esteem,	6376 children (4th grade primary school)
15	Lindow et al.	2020	Gordon / Caplan	Universal and Primary Prevention	USA	Mental health literacy	1878 adolescents (age 12–18)
16	López et al.	2020	Gordon	Indicated Prevention	Spain	Depression	173 working adults (non-professional caregivers)
17	Missler et al.	2020	Gordon	Universal Prevention	Netherlands	Anxiety, depression	234 adults
18	Streimann et al.	2020	Gordon	Universal Prevention	Estonia	Mental health, behavioral problems	708 children (1st grade primary school)
19	Braun et al.	2021	Gordon	Indicated Prevention	Germany	Depression, mental health	360 working adults (green professions)
20	Ebert et al.	2021	Gordon	Universal Prevention	Germany	Mental health, stress	396 working adults
21	Johnson and Wade	2021	Gordon	Universal Prevention	Australia	Anxiety, depression, stress, well-being	434 adolescents (8th & 10th grade)
22	O’Keeffe et al.	2021	Gordon	Universal Prevention	Ireland	Anxiety, depression, mindfulness, well-being	355 children (primary school)
23	Overbeek et al.	2021	Gordon	Indicated Prevention	Netherlands	Mental health, conduct problems	387 children (age 4–8)
24	Przybylko et al.	2021	Caplan	Primary Prevention	Australia, New Zealand	Mental health, emotional wellness	425 adults
25	Purgato et al.	2021	Gordon	Indicated Prevention	Italy, Germany, Austria, Finland, UK	Psychological distress, well-being	459 adults
26	Terry et al.	2021	Gordon	Selective Prevention	USA	Self-efficacy	43 adolescents
27	Auweiler et al.	2022	Caplan	Primary Prevention	Germany	Psychosocial risk	312 adults
28	Bhandari	2022	Caplan	Tertiary Prevention	India	Anxiety depression, PTSD, quality of life	72 adults
29	Bolinski et al.	2022	Gordon	Indicated Prevention	Netherlands	Anxiety, depression	35 university students
30	Ekbäck et al.	2022	Gordon	Indicated Prevention	Sweden	Anxiety, depression, stress	23 university students
31	Feinberg et al.	2022	Gordon	Universal Prevention	USA	Family resilience, mental health	399 families (parents and children)
32	Imamura et al.	2022	Caplan	Primary Prevention	Japan	Psychological distress	1200 working adults
33	Scalora et al.	2022	in association to Gordon	Targeted Prevention	USA	Anxiety disorder, depression	77 university students (age 18–24)
34	Deady et al.	2023	Gordon	Selective Prevention	Australia	Anxiety, burnout, depression	2084 working adults
35	Huber et al.	2023	Caplan	Primary Prevention	Austria, Germany	Mental health	69 adults (age 50–60)
36	Owens and Bunce	2023	in association to Gordon	Universal or targeted Prevention	UK	Stress, well-being	76 adults (age 18–25)
37	Baetens et al.	2024	Gordon	Universal Prevention	Belgium	Depression, mental health	329 adolescents (age 11–14)
38	Binder et al.	2024	Gordon	Indicated Prevention	Germany	Burnout, stress, psychosomatic symptoms	70 adolescents (age 13–18)
39	Rith-Najarian et al.	2024	Gordon	Universal Prevention	USA	Anxiety, depression	1607 university students
40	Teesson et al.	2024	Gordon	Universal Prevention	Australia	Anxiety, depression	6386 adolescents (age 13–20)

### Geographical Distribution and Types of Studies

The included studies collected data from 19 different countries across four continents (Asia, n = 5; Australia, n = 5; Europe, n = 22; North America, n = 8), with most of the research conducted in Germany (n = 8), the USA (n = 8), and the Netherlands (n = 6). Four studies collected data from two to five countries. There was no significant geographic differentiation in the application of the frameworks: Caplan’s framework was applied in five countries across three continents, whereas Gordon’s framework was applied in all 19 countries across four continents. Of the 40 studies, 30 were randomized controlled trials, and ten were clinical trials with various study designs.

### Time Trends

From 2018 to 2024, the application of Gordon’s framework was more frequent, particularly in universal (n = 15) and indicated prevention (n = 12). The peak of overall research activity occurred in 2020 and 2021, with eight studies (n = 8) conducted each year. Universal prevention was most frequently applied in 2020 (n = 4), whereas the indicated prevention reached its highest application in 2019 (n = 4). Caplan’s framework played a more secondary role but peaked in 2022, with three studies primarily applying primary prevention (n = 2). Research on tertiary prevention was only applied in one study by 2022.

### Dimensions of Mental Health

The included studies typically examined multiple dimensions of mental health within their interventions. In 34 studies, between two and five dimensions were addressed, whereas in six studies, a single dimension of mental health prevention was the primary focus. On average, each study examined 2.2 dimensions. It is essential to recognize that the boundaries between different dimensions of mental health are not always clearly defined and that one dimension may manifest as a symptom of another. For instance, stress and anxiety can be symptomatic of depression and may be integral to the concept of burnout (Bianchi et al., 2015; Craske et al., 2017). Table 2 presents the frequency with which each mental health dimension was investigated across all studies and the corresponding application of the framework.

**Table 2:** Observed dimensions of mental health

Prevention framework	Type of prevention	Depres-sion	Anxiety	Mental Health	Stress	Burn-out	Other dimensions
Caplan’s framework	Primary prevention			2			3
	Secondary prevention						
	Tertiary prevention	1	1				2
	Combination of primary and secondary	1	1		1		1
Gordon’s framework	Universal prevention	11	9	4	3		10
	Selective prevention	2	1			1	1
	Indicated prevention	8	7	2	3	1	4
In association with Gordon’s framework	Universal and targeted prevention				1		1
	Targeted prevention	2	2				1
Combination of both frameworks	Universal and primary prevention						1
	Total	25	21	8	8	2	24

Anxiety and depression were frequently assessed in studies applying Gordon’s universal and indicated prevention approaches, reflecting a focus on the general population as well as on risk groups and individuals exhibiting early symptoms. Stress, in contrast, was commonly addressed not only in universal and indicated prevention but also through a combination of primary and secondary prevention, emphasizing the promotion of coping strategies across both the general population and high-risk groups. Well-being and quality of life were explored across various studies, particularly those combining different prevention types or frameworks.

The diversity of dimensions and outcomes highlights the methodological variety across the included studies, illustrating how the frameworks were applied to address distinct mental health challenges.

### Demographic Characteristics of the Target Populations

This review analyzes 40 studies that span a diverse array of demographic groups. Sixteen studies focused on children and adolescents, with sample sizes ranging from 43 to 6,386 participants aged 4 to 20. Seven studies focused on children, eight examined adolescents, and one both. According to Gordon’s framework, most of these studies (n = 9) were classified as universal prevention.

University students were the subjects of six studies, of which the majority (n = 3) were classified as indicated prevention within Gordon’s framework. Sample sizes ranged from 23 to 1,607 participants; most studies involved mixed-gender groups and addressed subclinical symptoms of depression and anxiety. The age range in this category was 16 years, with no upper age limit specified in some studies.

Working adults were the primary focus of seven studies, with sample sizes ranging from 173 to 2,084 participants. There was no clear preference for any specific preventive framework in this group. These studies often targeted specific professions, such as healthcare workers, and those in green sectors, such as agriculture, horticulture, and forestry. Many studies in this category did not provide specific age ranges for participants.

Eight studies focused on adults without specifying further details. The sample sizes ranged from 69 to 954 participants, with no specific age range provided. Most of these studies (n = 3) applied Caplan’s primary prevention classification. Two studies focused on parents and their children or adolescents, with sample sizes of 349 parents and 327 adolescents (aged 12–15 years) and 399 families (parents and children with no specific age criteria). Both studies were classified as universal prevention within Gordon’s framework.

Older adults were the focus of one study, with a sample size of 21 participants. This study involved individuals aged > 60, with co-occurring medical conditions, and were classified as indicated prevention within Gordon’s framework.

Overall, the studies spanned a broad demographic spectrum, from children as young as four to older adults over 60. This diversity provides valuable insights into the different target groups for mental health interventions. Notably, Caplan’s framework was predominantly applied in studies targeting adults and working populations.

## Discussion

The present investigation highlights that Caplan’s and Gordon’s prevention frameworks are primarily applied in isolation despite offering complementary approaches to disease prevention. Of the 40 included studies, only one (Lindow et al., 2020) employed both frameworks simultaneously. In this study, universal and primary prevention were treated as equivalent, without explicitly addressing their conceptual distinctions, illustrating the challenge of differentiating between the two approaches. This lack of conceptual clarity, along with the tendency to apply the frameworks separately, limits their potential to fully leverage their complementary strengths in holistic prevention strategies.

Although Gordon’s framework was applied more frequently (n = 33) than Caplan’s (n = 6), this discrepancy does not necessarily indicate dominance or superiority. In our results, no consistent pattern emerged that would suggest a systematic preference for one framework over the other based on regional differences, demographic factors, or time trends. However, we observed a slight tendency in which broadly defined outcomes, such as overall mental health, were primarily assessed in primary and universal prevention approaches, whereas more specific outcomes, including anxiety, depression, and stress, appeared across nearly all prevention levels. Although distinct patterns emerged in outcome selection within each framework, this review did not assess intervention effectiveness. Therefore, no conclusions can be drawn regarding the comparative efficacy of these frameworks in improving mental health outcomes.

The choice of framework may also be influenced by familiarity within research communities and disciplinary traditions, potentially shaping its adoption in different fields. Gordon’s framework, for instance, aligns strongly with public health strategies that prioritize broad, population-level interventions. By categorizing individuals based on risk, it enables scalable prevention efforts, particularly in schools and community programs, where large groups can be targeted efficiently, but may benefit from greater specificity when applied to clinical contexts (Compton & Shim, 2020). In contrast, Caplan’s framework is more commonly applied in clinical and healthcare settings, where prevention efforts focus on more specific mental health outcomes and targeted interventions, and it may not explicitly address the broader social and environmental determinants of mental health. This distinction is further reinforced by the greater visibility of Gordon’s framework in public health literature, which may contribute to its broader adoption in research and practice (Fusar-Poli et al., 2021; Singh et al., 2022).

The application of these frameworks can influence the design and implementation of mental health prevention programs. At the same time, it is also possible that an intervention or study design was predefined, with the appropriate framework being selected afterwards. Understanding these dynamics is particularly relevant when considering how Gordon’s approach facilitates scalable interventions across different risk levels, ranging from broad population-based prevention to targeted efforts for individuals at increased risk or exhibiting early symptoms.

While this risk-based classification enables structured and scalable prevention efforts, it does not fully account for how symptom progression can fluctuate over time in mental health conditions. This is particularly relevant for individuals whose risk status may change, requiring flexible and adaptive prevention strategies. Here, Caplan’s disease stage-based model provides a complementary structure, organizing prevention along the course of illness development. By integrating both frameworks, prevention strategies can be designed to not only scale across populations but also dynamically respond to individual trajectories of mental health risk and symptom evolution, enhancing their effectiveness and adaptability (Fusar-Poli et al., 2021).

This scoping review shows that Caplan’s and Gordon’s frameworks are applied across diverse demographic groups without a clear pattern. Given that neither model is limited to specific populations, their widespread use is unsurprising. Their broad application further highlights their flexibility and adaptability.

The temporal analysis indicated a peak in research activity in 2020 and 2021, with a subsequent decline in 2022, potentially due to disruptions caused by the COVID-19 pandemic. The pandemic led to delays in non-COVID-19 clinical trials and a shift in research priorities, explaining the reduced publication output during this period (He et al., 2023; Lasch et al., 2022). Despite the decline, the pandemic emphasized the importance of mental health interventions, potentially driving future shifts toward integrating both Caplan’s and Gordon’s frameworks to address emerging global mental health challenges. This suggests that flexible prevention frameworks, such as those combining stage-specific and risk-targeted interventions, may be necessary to mitigate future crises.

### Potential for Synergistic Application

A key finding of this review is the limited integration of Caplan’s and Gordon’s frameworks, representing a missed opportunity to enhance mental health prevention strategies. While both models offer valuable perspectives, their combined application could create a more comprehensive and adaptable approach. Applying both allows for a more precise and needs-based intervention strategy, ensuring that prevention efforts are neither too broad nor too narrow. To illustrate the practical benefits of this integration, the following two examples demonstrate how these frameworks can be applied in different contexts. The first is a fictional workplace mental health program, showing how the models can be structured within an organizational setting. The second presents a real-world case from the RV Fit Mental Health Program, implemented by the German Pension Insurance of Central Germany (Stephan et al., 2024), highlighting how both frameworks can guide a structured behavioral prevention initiative.

### Example 1: Workplace Mental Health Prevention Program

In this scenario, a company implements a multi-tiered prevention strategy. While this example is hypothetical, workplace mental health prevention programs incorporating a variety of interventions, including multi-tiered approaches and digital tools, have been implemented (Arensman et al., 2023; De Angelis et al., 2020; LaMontagne et al., 2014). The strategy first categorizes employees using Gordon’s risk-based model (universal, selective, and indicated prevention) and then applies Caplan’s disease stage-based approach (primary, secondary, and tertiary prevention) to tailor interventions based on symptom severity. Universal prevention applies to all employees who gain access to a mental health app offering stress reduction tools such as mindfulness exercises and psychoeducation. The app includes a brief digital screening to assess general mental health. Employees without symptoms continue with primary prevention measures aimed at promoting mental resilience and preventing distress. If early symptoms emerge, employees proceed to secondary or tertiary interventions, which are introduced accordingly. The selective prevention category focuses on employees in high-stress roles, such as those with high workloads, frequent decision-making pressure, or irregular shifts. Instead of screening the entire workforce in-depth, these employees are prioritized for proactive screening and additional prevention measures based on their increased risk exposure. Those without symptoms remain in primary prevention, using general well-being resources similar to universal prevention. However, those showing early signs of stress or emotional strain receive secondary prevention, such as interactive cognitive-behavioral training, coaching sessions, or targeted support groups. If symptoms are more severe, tertiary prevention measures, including structured supervisor check-ins or workplace counseling, are provided. The indicated prevention category includes employees who already exhibit clear signs of mental distress, such as increased absenteeism, reduced performance, or self-reported psychological strain. These individuals are guided toward secondary prevention, such as structured coaching and mental health training, or tertiary prevention, which may involve professional counseling, rehabilitation programs, or workplace accommodations to support recovery.

This workplace prevention program demonstrates how Gordon’s framework enables structured risk stratification, allowing companies to flexibly allocate resources by addressing either the entire workforce or specific high-risk groups. Caplan’s model further refines these interventions by aligning them with symptom severity, ensuring that employees receive appropriate support, from general well-being promotion to targeted therapeutic measures. By integrating both frameworks, this approach balances scalability with precision, making it adaptable to different organizational needs and resource constraints while effectively supporting employees at various levels of risk and symptom progression.

### Example 2: Structured Prevention in Practice—The RV Fit Mental Health Program

A example of the integration of Caplan’s and Gordon’s frameworks is the RV Fit Mental Health Program, implemented by the German Pension Insurance of Central Germany (Stephan et al., 2024). This program demonstrates how Gordon’s risk-based classification is used to define different access pathways, while Caplan’s stage-based model ensures that the intervention aligns with participants’ symptom severity. By combining these frameworks, the program enables a structured and needs-based prevention strategy, ensuring that resources are efficiently allocated while addressing varying levels of mental health risk.

The program applies Gordon’s framework to categorize participants based on their level of risk. The indicated prevention pathway includes individuals whose rehabilitation request has been denied by the German Pension Insurance and who have a specific ICD-coded mental health diagnosis. These participants are identified through existing rehabilitation application data from German Pension Insurance Central Germany and are directly recruited into the program. The selective prevention pathway includes individuals identified by health insurance providers who have been on sick leave for 14 consecutive days due to specific ICD-coded mental health conditions. The universal prevention pathway is open to the general population and promoted through public outreach by clinics, allowing for voluntary participation.

Before entering the program, participants in the selective and universal prevention pathways undergo a screening process using a standardized screening instrument to ensure their symptom severity aligns with the program’s focus on secondary prevention. If participants are identified as suitable for primary or tertiary prevention during screening, they are referred to other programs offered by the German Pension Insurance.

Once participants are enrolled, the intervention is structured according to Caplan’s disease stage-based model. At the beginning of the program, a comprehensive needs assessment evaluates each participant’s prevention needs. Based on this assessment, participants collaborate with a therapist to establish personalized prevention goals. The program consists of a core set of prevention modules designed for all participants, supplemented by individually selected modules tailored to personal health goals and risk factors. As the intervention focuses on secondary prevention, it aims to prevent further deterioration, strengthen coping mechanisms, and promote long-term mental health resilience.

This approach highlights the complementary strengths of both frameworks. Gordon’s model ensures that prevention efforts effectively reach different risk groups, facilitating structured recruitment. Caplan’s model provides a framework for selecting appropriate prevention modules that correspond to participants’ symptom progression, ensuring that interventions are not generic but tailored to specific needs. By integrating risk-based classification with disease stage-based intervention design, the RV Fit Mental Health Program serves as a practical model for structured and scalable prevention strategies.

### Policy and Practical Implications and Future Research Directions

The examples illustrate how Caplan’s and Gordon’s frameworks can be effectively integrated to enhance mental health prevention strategies. The workplace mental health program demonstrates how a structured, risk-based approach can be implemented in an organizational setting, while the RV Fit Mental Health Program applies these frameworks in a real-world intervention, using risk classification for participant recruitment and symptom progression to guide individualized prevention planning. By combining Gordon’s risk stratification with Caplan’s structured intervention planning, these approaches enable a more precise, scalable, and needs-based prevention strategy. Gordon’s model ensures targeted prevention efforts, while Caplan’s framework provides a structured mechanism to tailor interventions to individual needs. This integration moves beyond generic prevention approaches, allowing for a more efficient allocation of resources and support that aligns with individual risk profiles and symptom severity.

From a policy perspective, an integrated approach can enhance resource allocation by maintaining universal interventions while using risk stratification to direct intensive support where needed. This is particularly crucial in resource-limited settings, where balancing broad outreach with specialized interventions is essential for equity and impact. In practice, aligning the two frameworks can strengthen existing prevention programs by enabling tiered services across health care, schools, and workplaces. This structure facilitates earlier detection, continuous monitoring, and follow-up, reducing the risk of chronicity or relapse.

Future research should examine how this combined application influences mental health outcomes across diverse settings and populations. Key areas of investigation include the long-term effectiveness of integrated prevention strategies, their cost-effectiveness compared to standardized prevention programs, and the scalability of these models in different healthcare and workplace environments. Studies employing longitudinal designs and economic evaluations could provide valuable insights into the sustainability and broader impact of these approaches.

### Limitations

This scoping review has limitations. First, the selection of included studies was restricted to specific databases and languages, which may have introduced a potential bias in the study selection process. The exclusion of Web of Science and other valuable databases is a limitation, as it may have resulted in the omission of relevant studies. However, the search covered key databases, including PubMed, Scopus, and EBSCOhost (including APA PsycArticles and PubPsych), which are widely recognized as essential sources for mental health research. This selection ensures broad coverage of relevant literature despite the exclusion of additional databases. Additionally, excluding grey literature and non-peer-reviewed studies may have limited the scope of our findings, as such sources could offer innovative or practical approaches to prevention, particularly in non-academic or community-based settings. However, this exclusion aligns with the primary aim of the scoping review, which was to synthesize evidence from peer-reviewed studies that meet established methodological standards. Peer-reviewed research ensures a baseline of scientific rigor and consistency, which is particularly important given that this review does not independently assess the quality of included studies, as per the scoping review methodology (Arksey & O’Malley, 2005; Peters et al., 2024). Future reviews or complementary studies could incorporate grey literature to capture a broader spectrum of evidence, particularly for real-world applications of prevention frameworks. Additionally, the review did not include a registered protocol, which limits transparency and reproducibility. However, the study adhered to established scoping review frameworks, ensuring methodological rigor.

## Conclusion

This investigation underscores the value of Caplan’s disease stage-specific approach and Gordon’s risk-targeted strategies as complementary frameworks for classifying preventive measures in mental health. Classifying prevention measures is crucial because it ensures that the right target groups are addressed and appropriate outcomes, such as effectiveness and impact, are measured. By distinguishing between disease stages and risk levels, prevention programs can be tailored to specific needs, leading to more effective and efficient health interventions. Additionally, classification applying Caplan’s and Gordon’s frameworks simultaneously enables comparability within interventions, where different pathways to prevention services can be categorized and between interventions, allowing for the evaluation and optimization of strategies across various populations and contexts. While both frameworks offer distinct benefits, their combined application has the potential to create more comprehensive prevention programs. However, existing literature shows that these frameworks are primarily applied in isolation, limiting the opportunity to realize their full synergistic potential.

To address this gap, future research should prioritize pilot studies that evaluate the practical integration of these frameworks. Developing hybrid interventions that blend Caplan’s focus on disease stages with Gordon’s risk stratification could lead to more tailored and effective prevention strategies. Mixed methods approach or randomized controlled trials would be ideal for assessing the feasibility and effectiveness of such integrated frameworks. Given the global challenges posed by crises like the COVID-19 pandemic, flexible and adaptable prevention strategies are essential. Future research should also explore potential barriers, such as resource allocation and coordination between population-wide and targeted approaches. By overcoming these challenges, researchers can develop prevention programs that cater effectively to both general populations and high-risk groups.

Integrating these findings into newly developed preventive interventions, such as the RV Fit Mental Health program, could further optimize interventions to promote and strengthen mental health. Tailoring prevention strategies to the specific needs of target populations can enhance the efficiency, comprehensiveness, and overall impact of prevention efforts, particularly during global crises.

## Data Availability

Not applicable.
